# An assessment study of CVD related risk factors in a tribal population of India

**DOI:** 10.1186/s12889-016-3106-x

**Published:** 2016-05-25

**Authors:** Vani Kandpal, M. P. Sachdeva, K. N. Saraswathy

**Affiliations:** Department of Anthropology, University of Delhi, Delhi, India

**Keywords:** Non-communicable diseases, Hypertension, Obesity, Metabolic syndrome, Fasting blood glucose

## Abstract

**Background:**

Non communicable diseases (NCDs) have become a major concern for global health. Cardiovascular diseases (CVDs) contribute 48 % towards the deaths due to NCDs in India. Though studies have been conducted in urban and rural areas, data related to tribal communities is limited. The present study aims to examine various CVD related risk factors including hypertension, elevated fasting blood glucose, obesity and metabolic syndrome among a tribal population.

**Methods:**

The present study was an observational, cross- sectional study conducted on Rang Bhotias, a tribal population of India. The participants were adults of age between 20 and 60 years. Prior to blood sample collection, interview schedule was administered which included relevant information like age, lifestyle, socio-economic status, education and occupation In addition to this, various anthropometric and physiological measurements were taken. Logistic regression was used to examine the association of the various health disorders related to CVDs with age, gender and behavioural factors (smoking, alcohol consumption and physical activity).

**Results:**

A total of 288 participants were surveyed for the study including 104 males and 184 females. High BMI (56.6 %), hypertension (43.4 %), metabolic syndrome (39.2 %) and abdominal obesity (33.7 %) were the most prevalent CVD risk factors observed in the population. The multivariate logistic regression analysis, conducted to examine the contribution of risk factors including behavioural risk factors on the studied abnormalities, revealed age to be a significant risk factor for all the abnormalities except elevated fasting blood glucose. Gender and physical inactivity contributed significantly towards development of hypertension. Physical inactivity was also found to be associated with high BMI levels.

**Conclusion:**

In the present study, hypertension, high BMI levels, MS and abdominal obesity have been found to be high among the studied population. The status of the population with respect to these abnormalities implicates susceptibility of the community towards various common disorders. The prevention and treatment intervention programs should be implemented taking into consideration age and gender.

**Electronic supplementary material:**

The online version of this article (doi:10.1186/s12889-016-3106-x) contains supplementary material, which is available to authorized users.

## Background

Today, millions of people in developing nations are facing double health burden of diseases associated with infection and nutrition along with the load of chronic non-communicable diseases (NCDs). Rapid urbanisation has led to changes in daily activity, diet and lifestyle leading to NCDs like diabetes, Cardiovascular Diseases (CVDs), neuropsychiatric disorders etc. Of the estimated 57 million global deaths in 2008, 36 million were due to NCDs [1]. Largest proportion of NCD deaths have been caused by CVDs (48 %) followed by cancers (21 %). Fifty percent of all deaths and 62 % of the total disease burden can be attributed to NCDs in India [2]. Cardiovascular disease, diabetes mellitus and stroke are the NCDs which have lately emerged as public health concern [3]. According to estimates, CVDs will be the largest cause of disability and death in India by 2020 [4]. Epidemiological studies suggest increased prevalence of CVD risk factors such as tobacco consumption, smoking, hypertension, diabetes, obesity and lipids in India. However, regional variations have been observed with respect to these factors [5]. Traditional CVD risk factors include hypertension, diabetes etc. [6]. Recently metabolic syndrome (MS), has been an addition in the risk factor category of CVDs. It is characterised by constellation of risk factors encompassing abdominal obesity, raised blood pressure, hypertriglyceridemia, depressed plasma HDL cholesterol and raised fasting blood glucose. Individuals with MS have been reported to be 3–10 times more likely to develop cardiovascular disease [7].

CVDs constitute the leading cause among NCDs in India [8]. Country such as India with enormous diversity, has huge variation in prevalence and risk factors of NCDs. Studies conducted in urban and rural settings regarding CVD risk factors have shown significant differences [5]. Within Indian context, tribal population, restricted to rural areas, are associated with poverty, illiteracy, malnutrition [9]. Thus, they are assumed to be untouched by NCDs which are lifestyle driven diseases. However, recent studies have produced evidence for increasing trend of NCDs among tribal population groups [9, 10]. ICMR conducted a survey among 7 states of India based on WHO’s STEPS method to investigate NCD risk in 2007-2008 [11]. Though these states are inhabited by tribal population, prevalence of NCDs were reported among urban, rural and combined population. Studies have been conducted on tribal population groups of different states including Mahrashtra, Gujarat, Andaman and Nicobar islands, Kerela and Karnataka on various risk factors associated with NCDs [12–17]. However, owing to their diverse ethnic background, culture, diet, habitat and behavioural habits, tribes are expected to have community specific risk factors. Very few studies [18–20] are available on tribes of hilly terrain of Himalayan belt. Since NCDs have become a public health challenge, surveillance of risk factors associated with CVDs, a leading cause among NCDs, in tribal communities is essential for developing prevention strategies and implementing control programmes.

Rang Bhotia is a tribal population residing in a relatively high altitude and is exposed to rough terrain and climatic conditions which are not found in plain areas. This is a tribe which is expected to be in epidemiological transition and remains untouched by the research fraternity. As a result no data is available regarding CVD risk factors on this population. Thus, the present study attempts to examine various CVD risk factors including hypertension, elevated fasting blood glucose, obesity and metabolic syndrome among this tribal population of Uttarakhand, India.

## Methods

### Study design and setting

The present study was an observational cross sectional study. The participants constituted individuals belonging to a tribal population of India. Participants were recruited in the study through purposive sampling. The study was approved by the ethical committee of the Department of Anthropology, University of Delhi and individuals participated in the study with informed written consent. The incharge of the local tribal welfare committee was contacted before the commencement of the study and oral consent was obtained to conduct the study.

### Study population and inclusion criteria

Bhotias of Uttarakhand state have a population of 36,438 as per census 2001. Bhotiyas are not ethnically as well as culturally homogenous [21]. They are divided into various subgroups which don’t intermarry among themselves. Rang Bhotia is one such subgroup which inhabits Dharchula in Pithoragarh district of Uttarakhand state. Though the size of this population is not available (as its a subgroup), but as per census 2011 population of scheduled tribe in the tehsil Dharchula constituting Rang Bhotia and a primitive tribal group (i.e. Raji) is nearly 7308. They show close affinity with Tibetans physically as well as culturally [22].

Endogamous population of Rang Bhotia is confined to Pithoragarh District of Uttarakhand and within the district it is restricted to Dharchula tehsil, a subdivision of district with 60 villages. The present study was conducted in 12 villages of Dharchula where size of this population group is relatively higher. household survey was conducted to identify individuals belonging to this tribal community and they constituted sampling frame. Individuals unrelated upto first cousin were included as the study extends to inclusion of the genetic variables, which are not in the purview of this manuscript. Both males and females of age group between 20 and 60 years were recruited in the study. Individuals above 60 years of age, pregnant ladies and disabled persons of the considered age group were excluded from the study. We calculated sample size assuming prevalence of hypertension as 11.1 % among a previous study on a Himalayan tribe (inhabiting rural area) since no data was available on the selected population [20]. Sample size was found to be 152 with 95 % confidence coefficient.

### Data collection

Prior to sample collection, interview schedule was administered on recruited individuals after prior informed written consent. Interview schedule included relevant information like age, lifestyle, socio-economic status, education and occupation. Smokers constituted individuals who were currently smoking while non smokers included who havent’ smoked ever or have been ex smokers for the past 1 year. Consuming alcoholic beverage was considered as drinker and included as a risk factor. Physical activity was self reported and were classified as - sedentary and active (Participants with predominantly sedentary routiene were considered sedantary while participants with predominantly standing, walking or physically strenuous routiene were categorised as active). Anthropometric measurements such as weight, height and waist circumference were measured. Subjects were informed before hand for blood sample collection and after overnight 12 h fasting, blood samples were collected. Fasting Blood sugar was measured by glucometer. The blood sample was collected in a plain test tube for the measurement of fasting lipids (triglyceride, total cholesterol and high density lipoprotein-cholesterol (HDL-C). All blood samples were properly labelled and transported to a private certified laboratory for analysis. Lipid analysis was performed using Randox assays for the respective lipids on spectrophotometer (Additional File 1 contains recorded data). In every batch of samples (given 5 times totally) given in laboratory, for quality assurance five samples were put in duplicates and their respective measurements were cross checked.

### Measurement of blood pressure and anthropometric variables

Blood pressure was measured in the right arm of the participant in sitting position using mercury sphygmomanometer. Mercury Sphygmomanometer is a gold standard to measure BP. Before using the instrument, intra and inter rater reliability was checked on 20 individuals. The average value of the measurements of two readings taken at an interval of 5 min was used for the analysis. Waist circumference was measured over light clothing at a level midway between the lower rib margin and iliac crest.

### Criteria and definitions used

#### MS assessment

In the present study, definition proposed by NHLBI [23] was used in assessing MS. It requires at least three of the following components: (i) abdominal obesity (waist circumference ≥90 cm for Asian men or ≥80 cm for Asian women), (ii) triglycerides ≥150 mg/dL, (iii) HDL cholesterol ≤40 mg/dL for men or 50 mg/dL for women, (iv) systolic/diastolic blood pressure ≥130/85 mmHg or receiving drug treatment, and (v) fasting plasma glucose ≥100 mg/dL.

#### Hypertension

According to JNC VII [24] on the basis of Systolic blood pressure (SBP) and Diastolic blood pressure (DBP), individuals can be categoried as Hypertensives when SBP ≥140 or DBP ≥90 mmHg.

#### Obesity

As per Asian guidelines, Overweight is defined by BMI: 23–24.9 Kg/m^2^ and obesity is defined by 25 Kg/m^2^ and above. High BMI levels included overweight and obesity category. Abdominal obesity was defined as waist circumference of ≥90 cm for men and ≥80 cm for women [25].

#### Elevated fasting blood glucose

Individuals with a fasting plasma glucose of ≥126 mg/dl or on medications for high blood sugar were considered to be having elevated fasting blood glucose (FBG).

#### Statistical analysis

Descriptive statistics were generated using cross-tabulations. Chi-square test was used to examine the association between categorical variables. Multivariate Logistic regression analysis was performed to simultaneously evaluate the effects of age, gender, alcohol consumption, smoking, physical activity (independent variables) on hypertension, obesity, elevated FBG and MS (dependent variables). A significance level of 5 % was used for all of the statistical tests. The data was analyzed using SPSS Version 16.0 (SPSS Inc., Chicago, IL, USA).

## Results

The present study included two hundred eighty eight (288) participants, which included 104 males and 184 females. The age of the subjects ranged from 20 to 60 years with mean age of the subjects being 41.3 ± 11.04 years. The mean age of males and females were observed to be 42.5 ± 12.03 years and 40.8 ± 10.42 years respectively. The general characteristics of the population were observed (Table 1). In the present study, 71.1 % of the individuals were found to be literate. Alcohol consumption was observed to be among 37.5 % of the participants and 78.8 % of males being alcohol consumers. While only 14.1 % of females consumed alcohol. The present study observed distribution of smokers to be 13.9 % which included 23.1 % of males and 8.7 % of females who smoked beedi/cigarettes. 82.3 % of the study participants were physically active which includes 82.7 % of males while 82.1 % of females.

**Table 1:** General Characteristics of the population

Characteristics		Males (*N* = 104)	Females (*N* = 184)	Total (*N* = 288)
Age (years)	20–34	32 (30.8 %)	44 (23.9 %)	76 (26.4 %)
	35 and above	72 (69.2 %)	140 (76.1 %)	212 (73.6 %)
Education	Illiterate	6 (5.8 %)	77 (41.8 %)	83 (28.8 %)
	Primary	21 (20.2 %)	37 (20.1 %)	58 (20.1 %)
	High School and above	77 (74 %)	70 (38 %)	147 (51 %)
Smoking	Smokers	24 (23.1 %)	16 (8.7 %)	40 (13.9 %)
	Non-Smokers	80 (76.9 %)	168 (91.3 %)	248 (86.1 %)
Alcohol Consumption	Drinker	82 (78.8 %)	26 (14.1 %)	108 (37.5 %)
	Abstainer	22 (21.2 %)	158 (85.9 %)	180 (62.5 %)
Physical activity	Active	86 (82.7 %)	151 (82.1 %)	237 (82.3 %)
	Sedantary	18 (17.3 %)	33 (17.9 %)	51 (17.7 %)

High BMI (56.6 %), Hypertension (43.4 %), MS (39.2 %) and abdominal obesity (33.7 %) were the most prevalent CVD risk factors observed in the population (Table 2). High BMI (57.6 %) followed by MS (40.2 %), abdominal obesity (36.4 %) and hypertension (35.3 %) were the major risk factors among females in the studied population. On the other hand, hypertension (57.8 %) followed by high BMI levels (54.8 %), MS (37.5 %) and abdominal obesity (28.8 %) were found to be higher among males. Males had significantly higher prevalence of hypertension. All the risk factors studied were significantly higher in the age group of 35 years and above. In the present study, only 6.9 % individuals were found to be having elevated FBG. Males had higher prevalence of elevated FBG (8.6 %) as compared to females. All the CVD risk factors were found to be much higher among individuals who were sedentary but significant differences was found only in case of hypertension and high BMI (Table 2).

**Table 2:** Distribution of participants in different categories of risk factors

General characteristics		HTN %	Elevated FBG %	Obesity %		MS %
				High BMI	Abdominal obesity	
Gender	Male	57.7	8.6	54.8	28.8	37.5
	Female	35.3*	6	57.6	36.4	40.2
	Total	43.4	6.9	56.6	33.7	39.2
Age	20–34	28.9	1.3	40.8	22.3	25
	35 ≤	48.5*	8.9*	62.3*	37.7*	44.3*
Smoking	Smokers	40	7.5	47.5	27.5	30
	Non-Smoker	43.9	6.8	58.1	34.6	40.7
Alcohol consumption	Drinker	50	9.2	51.8	25.9	34.2
	Abstainer	39.4	5.5	59.4	38.3	42.2
Physical Activity	Active	39.2	5.9	53.6	31.2	37.1
	Sedantary	62.7*	11.7	70.6*	45.1	49

*- *p*-value <0.05

The multivariate logistic regression analysis was conducted to examine the contribution of risk factors including behavioural risk factors (smoking, alcohol consumption and physical activity) on the studied abnormalities (Table 3). Males were found three times more susceptible towards hypertension (O.R = 3.79, 95 % C.I = 1.87–7.68) as compared to females at a significant level (*p* < 0.05). Age was found to be a significant risk factor with individuals of 35 years and above group being at almost three fold risk (O.R= 2.89, 95 % C.I.=1.58-5.29) in case of hypertension. Sedentary individuals were also at three fold risk (O.R = 2.84, 95 % C.I.=1.46-5.52) in developing hypertension at significant level (*p* < 0.05). In case of FBG, individuals with age 35 years and above were found to be at seven fold risk with borderline significance (O.R = 7.46, 95 % C.I. = 0.98–57.28, *p* = 0.053). Along with age (O.R = 2.61, 95 % C.I = 1.50–4.54), physical activity (O.R. = 2.07, 95 % C.I. = 1.06–4.05) was observed to be significantly associated with high BMI (*p* < 0.05). Age was also found to be significantly associated with abdominal obesity (*p* < 0.05). Participants of age group 35 and above were found to be at two fold risk of developing abdominal obesity (O.R. = 2.24, 95 % C.I. = 1.20–4.10). In case of MS, age was found to be contributing significant risk (O.R. = 2.58, 95 % C.I. = 1.42–4.71) towards its development.

**Table 3:** Multiple Logistic Regression Analysis

General characteristics		HTN O.R. (95 % C.I.)	Elevated FBG O.R. (95 % C.I.)	Obesity O.R. (95 % C.I.)		MS O.R. (95 % C.I.)
				High BMI	Abdominal obesity	
Gender	Female	Reference	Reference	Reference	Reference	Reference
	Male	3.79 (1.87–7.68)*	1.27 (0.77–6.07)	1.36 (0.69–2.66)	1.13 (0.56–2.24)	1.35 (0.69–2.63)
Age	20–34	Reference	Reference	Reference	Reference	Reference
	35 and above	2.89 (1.58–5.29)*	7.46 (0.98–57.28)	2.61 (1.50–4.54)*	2.24 (1.20–4.10)*	2.58 (1.42–4.71)*
Smoking	Non Smokers	Reference	Reference	Reference	Reference	Reference
	Smoker	0.62 (0.29–1.33)	0.82 (0.21–3.14)	0.66 (0.32–1.37)	0.84 (0.38–1.85)	0.64 (0.30–1.38)
Alcohol consumption	Abstainer	Reference	Reference	Reference	Reference	Reference
	Drinker	0.74 (0.36–1.50)	1.54 (0.47–4.98)	0.63 (0.32–1.37)	0.52 (0.25–1.05)	0.61 (0.31–1.21)
Physical Activity	Active	Reference	Reference	Reference	Reference	Reference
	Sedentary	2.84 (1.46–5.52)*	2.17 (0.77–6.07)	2.07 (1.06–4.05)*	1.78 (0.94–3.34)	1.60 (0.85–2.99)

*- *p*-value <0.05

## Discussion

In the present world even tribal communities haven’t remained unaffected from urbanisation, which has led to changes in their lifestyle. Thus, health assessment of these communities is of prime importance. The present study observed more than 40 % participants being affected by hypertension. It’s a major health concern in developing nations and an important modifiable risk factor for CVDs. Studies among tribals have also reported hypertension among them. Tribes of western India had hypertension ranging from 16 to 30 % [13, 26]. Nicobarese an aboriginal tribes had a higher prevalence (50 %) according to a study conducted few years back [12]. Studies conducted on two tribal populations of South India reported high variance with 21.7 % hypertensives in one group while 40 % hypertensives in another group [14, 15]. Another study on tribals of central India observed prevalence of hypertension to be 23 % [16]. Studies have revealed acculturation being one of the reason for increased hypertension among tribals [10, 27]. Moreover, high alcohol consumption among males (78 %) can also explain the status of hypertension in the population.

India is the unofficial ‘diabetes capital of the world’ with an estimated 40 million affected and the numbers expected to reach 80 million by 2030 [28]. A meta analysis on seven studies on tribal population revealed the prevalence of diabetes to be 5.9 % [29]. A recent study among a Himalyan tribe inhabiting the native area and urban area observed prevalence of elevated FBG to be 3.9 % and 7.8 % respectively [20]. The present study, reporting 6.9 % to be elevated FBG, corroborates with the results of the other studies conducted in this respect. This might be due to change in their indigenous lifestyle by adopting new modern lifestyle.

Obesity and overweight have become a pandemic lately. The prevalence of obesity in urban area is higher as compared to rural in India [30]. As far as tribals are concerned, they have been observed to be undernourished in [9, 31]. Two mongloiod tribes, residing adjacently, Toto and Bhutia, showed wide disparities w.r.t high BMI values (BMI ≥28.8 kg/m2) with Toto showing 0 %, while Bhutia were reported to have prevalence of be 17 % and 9.3 % among urban Bhutia and rural Bhutia repectively [18]. It can be suggested that their contact with modernisation is leading to obesity among tribals. A tribe of western India showed prevalence of overweight and obesity to be 10.9 and 15.8 %, respectively [32]. Another study among Nicobarese showed 37 % to be overweight/obese [12]. Though the present study observed nearly 80 % of physically active participants, high levels of abnormal BMI (56.6 %) among them  is a major health concern.

High prevalence of abdominal obesity (34 %) was observed in the studied tribal populations. Among Asians, abdominal obesity has been reported to be high [33]. While Toto had 0.75 % abdominal obesity, rural Bhutia had 10.6 % in the total population [18]. A study on tribe of Western India reported 22.5 % individuals being abdominally obese [32]. On the other hand, a tribe from eastern India revealed 11 % to be affected with central obesity [17]. Unlike other studies, a study among Himalayan tribe reported 43.3 % to be centrally obese [20]. Asians have been observed to be predisposed to central obesity [20]. Moreover, the association of short statured population with abdominal obesity might be the reason for high abdominal obesity in the present population [34].

In the present study, prevalence of metabolic syndrome was found to be, according to NHLBI definition, 39.2 %. Different studies in India have reported prevalence ranging from 6 % to 51.4 % [18, 35–37]. The reason for the existence of such a wide range of prevalence can be attributed to different criteria employed, different age groups and different population groups. A study was conducted among two mongoloid tribal populations of India, Toto and Bhutia. Both showed marked differences on the basis of prevalence of metabolic syndrome. Bhutia were reported to have prevalence approximately of 42 %whereas Toto had prevalence as low as nearly 6 % [18]. A study on Kodavas, an indigenous group of Karnataka state, reported prevalence of MS to be 60 % [38]. Another study among a tribe of South India revealed prevalence to be 28.3 % [39]. Thus, it can be concluded that this syndrome is not necessarily confined to urbanised world. More studies need to be conducted to present the actual scenario.

Studies have shown gender to be a significant contributor for hypertension. Males have higher blood pressure than females through major part of their life, globally and only after age of 70 females have higher prevalence of hypertension. Thus, most of the epidemiological data report higher prevalence of hypertension among males [40]. Similarly, our study reports higher prevalence among males with three fold risk to develop hypertension. Apart from gender, age was also found to be associated. A study including tribes from nine states reported age to be significantly associated with hypertension [41]. In addition to age, physical inactivity has been found to be associated with hypertension [42]. Intervention studies have also shown that increased physical activity lowers blood pressure [43]. Alike the present study, many studies have also revealed significant association of physical inactivity with hypertension [44–46].

A major concern among the Indians is onset of diabetes at a younger age. Indians get diabetes earlier than their Western counterparts [47]. The present study observed individuals, with age 35 years and above, to be at seven times more risk for elevated FBG. Since the odds ratio had broad confidence intervals and was borderline significant, studies with large sample size might validate the results.

Advancing age was found to be an important factor for higher BMI levels. Many studies have revealed body mass index (BMI) to be associated with increasing age [48–50]. A study on males of one of the Naga tribes also reported increasing age being associated with BMI [51]. The present study also reported age to be associated with the abnormal BMI (overweight/obese) as well as abdominal obesity. The effect of age on abdominal obesity has been reported to be confounded by overall obesity [52]. As far as relation between BMI and physical activity is concerned, low physical activity has been associated with obesity as per WHO report. Physical activity has been observed to reduce the gains in BMI [53]. The present study reported significant association of the subjectively assessed physical activity with high BMI. Detailed information regarding intensity of physical activity could have revealed true association.

Age was found to be significantly associated with development of MS in the present study. Several studies across the world, among the Chinese, Americans, Inuits, Europeans and Finnish population, have reported age to be an important risk factor [54]. Age was reported to be an important risk factor in a North Indian study where less than 35 years of age group individuals had less prevalence of MS than above 35 years [35]. Another study among Kodavas, reporting increase in prevalence of MS with age, corroborates with the results of present study [38].

Increased prevalence of hypertension, which might be an adaptive strategy for this population residing at relatively high altitude and central obesity among the population clearly indicates high prevalence of MS, which has already been observed in the study. Association of physical inactivity with BMI levels and hypertension, in this physically active population, is a matter of concern. This suggests physical activity being an important predictor of lifestyle diseases in the population and awareness needs to be spread regarding its importance in the community. Studies with large sample size might validate the finding.

The present study attempted to provide a snapshot in context of the health status of the tribal population, but a large sample size would have led to affirmed results. Difficult terrain and harsh climate proved a hurdle for collecting large samples. The study had unequal male- female proportion which can be attributed to non-participation of potential male participants. Detailed information regarding alcohol, smoking and physical activity could have led to better insight regarding risk associated with studied health outcomes. Despite these limitations, the present study is not of less importance. The present study is among one of the few studies on a tribal population and very first study to be conducted on this endogamous hill population focussing various CVD related risk factors. Though small sample size ia a limitation but for homogenous population smaller sample sizes are adequate as compared to studies on heterogenous population [55].

## Conclusion

The present study observed elevated BMI levels, hypertension, MS and abdominal obesity to be high among the studied population group. Age was found to be significantly associated with the studied risk factors except elevated FBG. In addition to age, while gender and physical activity were found contributing significantly towards hypertension, on the other hand physical inactivity was found to be a risk factor for high BMI levels. Thus, awareness needs to be spread among the community regarding importance of physical activity. The status of the tribal population with respect to these health outcomes implicates susceptibility of the community towards CVDs. Drastic changes in various aspects of the life of tribal people, with their traditional ways of living, might make them more prone towards these lifestyle disorders. Combating CVDs, a major public health challenge among developing nations, requires intervention programmes including lifestyle and diet modifications. Identifying the community specific risk factors might help in implementation of health programmes at grass root level leading to reduction in the common disorders among the tribal population.

## Additional file

Additional file 1: Survey of CVD Related Risk Factors. (CSV 15 kb)
